# Biomolecular network querying: a promising approach in systems biology

**DOI:** 10.1186/1752-0509-2-5

**Published:** 2008-01-18

**Authors:** Shihua Zhang, Xiang-Sun Zhang, Luonan Chen

**Affiliations:** 1 Academy of Mathematics and Systems Science, Chinese Academy of Sciences, Beijing 100080, China; 2 Graduate University of Chinese Academy of Sciences, Beijing 100049, China; 3 Institute of Systems Biology, Shanghai University, Shanghai 200444, China; 4 Department of Electrical Engineering, Osaka Sangyo University, Osaka 574-8530, Japan; 5 ERATO Aihara Complexity Modelling Project, JST, Tokyo 153-8505, Japan; 6 Institute of Industrial Science, University of Tokyo, Tokyo 153-8505, Japan

## Abstract

The rapid accumulation of various network-related data from multiple species and conditions (e.g. disease versus normal) provides unprecedented opportunities to study the function and evolution of biological systems. Comparison of biomolecular networks between species or conditions is a promising approach to understanding the essential mechanisms used by living organisms. Computationally, the basic goal of this network comparison or 'querying' is to uncover identical or similar subnetworks by mapping the queried network (e.g. a pathway or functional module) to another network or network database. Such comparative analysis may reveal biologically or clinically important pathways or regulatory networks. In particular, we argue that user-friendly tools for network querying will greatly enhance our ability to study the fundamental properties of biomolecular networks at a system-wide level.

## Background

With the rapid accumulation of 'omic' data from multiple species [1], various models of biological networks are being constructed, such as protein-protein interaction (PPI) networks [2,3], gene regulatory networks [4,5], gene co-expression networks [6-8], transcription regulatory networks [9], and metabolic networks [10,11]. Instead of looking at individual components, studies on those molecular networks provide new opportunities for understanding cellular biology and human health at a system-wide level. Because of the complexity of life, revealing how genes, proteins and small molecules interact to form functional cellular machinery is a major challenge in systems biology. Recent studies have made great progress in this field, which considerably expanded our insight into the organizational principles and cellular mechanisms of biological systems. For example, new insights have been gained regarding topological properties [10-12], modular organization [13], and motif enrichment [14]. In particular, network centrality and connectivity measures have been applied to identify essential genes in lower organisms [15] and cancer-related genes in humans [16].

Biological systems differ from each other not only because of differences in their components, but also because of differences in their network architectures. A complicated living organism cannot be fully understood by merely analyzing individual components, and it is the interactions between these components and networks that are ultimately responsible for an organism's form and function. For example, humans and chimpanzees are very similar on the sequence and gene expression level, but show striking differences in the "wiring" of their co-expression networks [17]. It is essential to address the similarities and differences between molecular networks by comparative network analysis, to find conserved regions, discover new biological functions, understand the evolution of protein interactions, and uncover underlying mechanisms of biological processes.

In this article, we will discuss the computational problem posed by biomolecular network querying, that is, mapping nodes (such as proteins or genes) of one network of interest (for example a complex, a pathway, a functional module, or a general biomolecular network) to another network or network database for uncovering identical or similar subnetworks. Automated querying tools for implementing such a network comparison will be essential for harnessing the information present in multiple networks across different species or across different conditions.

## Tools for identifying conservation between networks

**Figure 1:**
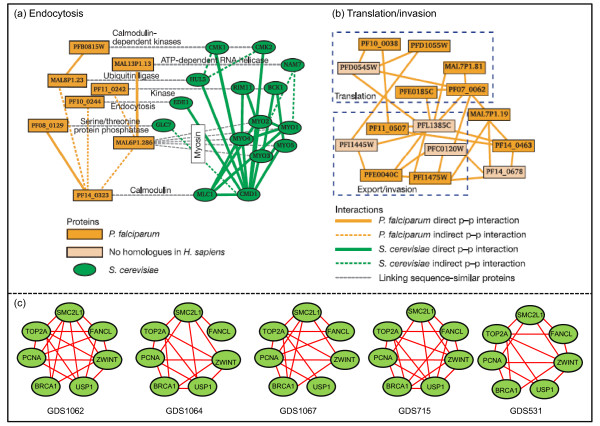
**Biomolecular network querying examples for multi-species and multi-conditions**. (a) A conserved complex identified between *Plasmodium falciparum *and *Saccharomyces cerevisiae*. (b) A representative complex uncovered within the *Plasmodium falciparum *network only. (c) A potential transcription module appeared in five leukemia gene co-expression networks under different conditions. Figures (a) and (b) were adopted by permission from Macmillan Publishers Ltd:  [31], copyright 2005, and figure (c) was redrawn from [33].

To provide an idea of the kind of tools that will be needed, we briefly review some recent advances regarding the identification of subnetworks or regions that are conserved within or across species [18-30]. One example is the PathBlast software developed by Trey Ideker's group [20-22], which allows one to compare protein interaction networks. By using PathBlast to compare multiple networks across different species, Suthram *et al*. [31] explored whether the divergence of *Plasmodium *at the sequence level can be embodied at the level of the structure of its protein interaction network. They found that *Plasmodium *has only three conserved complexes versus yeast, and no conserved complexes against fly, worm and bacteria. But yeast, fly and worm share an abundance of conserved complexes with each other. Figure 1(a) shows one of those three conserved complexes, which has a conserved counterpart in yeast, whereas Figure 1(b) is an example of a complex in *Plasmodium *without any conserved subnetworks to other organisms. Among the three conserved complexes, it has also been found that one protein in *Plasmodium *often has multiple homologous proteins in yeast, such as MAL6P1.286 in Figure 1(a). All these comparative results show that although there are a few similar substructures, the protein interaction networks between *Plasmodium *and the other four eukaryotes are considerably different, which implies different evolutionary processes in these species. Although there is a problem of reliability due to noise, the preliminary functional differences and underlying principles are worthy of further investigation.

A second example is MNAligner [29], developed by our group, which is an alignment tool for general biomolecular networks that combines both molecular similarity and topological similarity. This method can detect conserved subnetworks in an efficient manner without requiring special structures on the querying network. Another area of significant progress is multiple network alignment tools, e.g. Grælin developed by Flannick *et al*. [30], which uses a probabilistic function for topology matching, and can be applied to search for conserved functional modules among multiple protein interaction networks. Finally, using microarray data from multiple conditions and species, various comparative studies have been conducted so as to reveal transcriptional regulatory modules, predict gene functions, and uncover evolutionary mechanisms [32]. For example, Yan *et al*. [33] have developed a graph-based data-mining algorithm called NeMo to detect frequent co-expression modules among gene co-expression networks across various conditions. They found a large number of potential transcriptional modules, which are activated under multiple conditions. Figure 1(c) illustrates a condition-specific module that appears in five leukemia co-expression networks across different conditions. Moreover, genes in the module were found to be involved in the cell cycle and DNA repair, which is consistent with the nature of leukaemia; this gives an initial confirmation of the effectiveness of such an analysis.

## Tools for network querying

In addition to the studies on network comparison discussed above, a closely related technique is increasingly attracting attention and is expected to become a major analytical tool for systems biology. This technique is querying a small network against a large-scale network or a database of large-scale networks. Querying a small network is a local network comparison problem, which requires a highly efficient algorithm because it is computationally demanding. This problem has been studied by several groups [22,23,34,35], and a few search tools have been developed. However, the existing methods for querying are far from perfect, lagging behind the demands of the systems biology community.

For instance, although PathBLAST [20,22] can implement query searches, it is mainly only applicable to small pathways – up to 5 proteins – mainly due to the dimensionality problem with pathway length, and has limited support for identifying non-exact pathway matches. MetaPathwayHunter [23] developed by Pinter *et al*. enables fast queries for smaller pathways but is limited to those that take the form of a tree (i.e. a subnetwork with no loops). QPath [34] has also been developed for searching for linear pathways. Rather than finding networks with feedback loops, the algorithm mainly searches efficiently for homologous pathways, allowing for insertions and deletions of proteins in the pathways. NetMatch [35] is based on a graph-matching algorithm that aims to find the correspondences between two graphs. The results of NetMatch are subgraphs of the original graph connected in the same way as the querying graph, and therefore they can be viewed as candidate network motifs as a result of their similar topological features [14]. It can also handle multiple attributes per node and edge, but is impeded by the restrictive match requirement, i.e. one-one match without gap.

**Figure 2:**
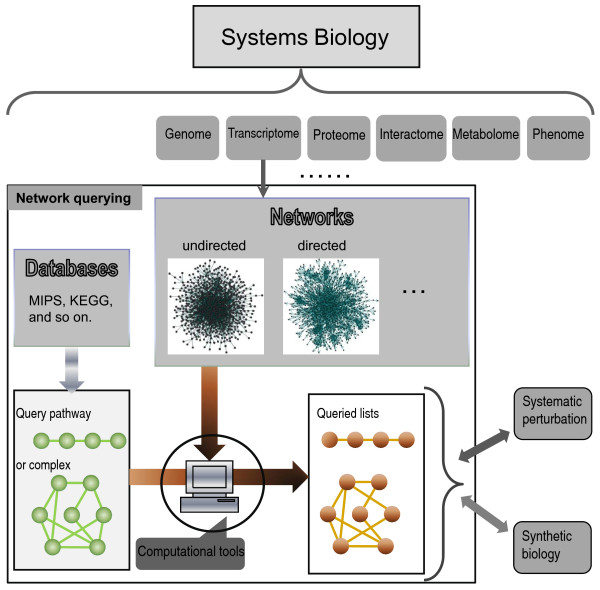
**Overview of biomolecular network querying from the perspectives of systems biology**. One major task for systems biology is to integrate information from genome (DNA) to phenome (phenotype) to predict mathematical models [38], which can then be tested by so-called 'synthetic biology' and/or system perturbations. The querying problem could be extended to various levels of '-omic' data and would then uncover more informative models of cellular mechanisms.

In addition to exploring networks, many querying tools, such as BLAST for sequence querying and DALI for structure querying, have been developed by researchers in other areas of computational biology, and have had a tremendous impact on the development of biological science. By analogy, given the growth in 'omics' or network-related databases (e.g. KEGG), network or pathway querying is expected to greatly enhance the research activity of systems biology (see Figure 2). For example, it would be useful if researchers constructing a portion of a pathway related to a disease of interest by analysis and integration of various experimental data could uncover the underlying biological processes involved in the disease by querying the 'pathway' in a pathway database.

## Future prospects for network querying and comparison

Computational techniques for network querying are obviously still at an early stage and are currently limited by several problems, such as computational complexity and simple topological structures. Like the querying methods for sequences, a universal querying system that can query a network (e.g. a protein complex, a pathway, a functional module, or a general biomolecular network) efficiently against a large-scale complicated network or a large-scale network database is very much needed. By exploiting the growing amount of information on complexes, functional modules and network motifs, one can transfer biological knowledge (e.g. functional annotations or missed interactions) to the subnetwork of another species, thereby increasing the information retrieved from noisy data.

Conventional querying tools generally aim at one specific 'type' of network, such as protein interaction networks, gene co-expression networks, metabolic networks or drug-target networks. Querying several different types of network can uncover more conserved functional units supported by integrated information. If we obtain an interesting pathway that exists in several co-expression networks under different conditions for one species, it clearly implies that the pathway is activated under several different conditions. On the other hand, if the querying is done among networks across different species, the uncovered subnetworks and the queried small network may provide valuable evolutionary information. We believe that evolution-based principles are crucial for network querying, just as substitution matrices and sequence evolution are important for sequence comparisons [36]. The noise and incompleteness of various 'omic' data are another important factor when we design such computational tools.

To benefit from the accumulation of network data, it will be important to develop user-friendly systems biology tools for biomolecular network querying. Recent advances in the field inspired by developments in sequence/structure alignment and large-scale database searching demonstrate the great potential of network querying in elucidating network organization, function and evolution. With the accumulation of huge network-related datasets, advances in computational methods and powerful software tools are being made possible by interdisciplinary cooperation across biology, physics, computer science and applied mathematics. With the development of powerful and sophisticated network querying tools, we expect to gain deep insight into essential mechanisms of biological systems at the network level from the perspective of systems biology.

## Authors' contributions

SZ proposed the main idea and drafted the manuscript. XSZ and LC gave valuable suggestions. All authors wrote and approved the manuscript.
